# Staphylococcus aureus Arsenal To Conquer the Lower Respiratory Tract

**DOI:** 10.1128/mSphere.00059-21

**Published:** 2021-05-19

**Authors:** Mariane Pivard, Karen Moreau, François Vandenesch

**Affiliations:** a CIRI, Centre International de Recherche en Infectiologie, Université de Lyon, Inserm, U1111, Université Claude Bernard Lyon 1, CNRS, UMR5308, ENS de Lyon, Lyon, France; b Centre National de Référence des Staphylocoques, Institut des agents infectieux, Hospices Civils de Lyon, Lyon, France; University of Nebraska Medical Center

**Keywords:** *Staphylococcus aureus*, virulence factors, community-acquired pneumonia, physiopathology, immune response, community-acquired infections, pathophysiology, pneumonia

## Abstract

Staphylococcus aureus is both a commensal and a pathogenic bacterium for humans. Its ability to induce severe infections is based on a wide range of virulence factors. S. aureus community-acquired pneumonia (SA-CAP) is rare and severe, and the contribution of certain virulence factors in this disease has been recognized over the past 2 decades. First, the factors involved in metabolism adaptation are crucial for S. aureus survival in the lower respiratory tract, and toxins and enzymes are required for it to cross the pulmonary epithelial barrier. S. aureus subsequently faces host defense mechanisms, including the epithelial barrier, but most importantly the immune system. Here, again, S. aureus uses myriad virulence factors to successfully escape from the host’s defenses and takes advantage of them. The impact of S. aureus virulence, combined with the collateral damage caused by an overwhelming immune response, leads to severe tissue damage and adverse clinical outcomes. In this review, we summarize step by step all of the S. aureus factors implicated in CAP and described to date, and we provide an outlook for future research.

## INTRODUCTION

Staphylococcus aureus is detected in 30% of the population, mostly as a nasal commensal, but also in the throat, on the skin, and in the gastrointestinal tract (1). In addition to these innocuous interactions with the human host, S. aureus has the potential to develop a wide range of diseases in humans, from mild infections of the skin and soft tissues to severe and fatal infections, such as bacteremia and pneumonia (2).

S. aureus pneumonia can be divided into two categories, hospital-acquired pneumonia (very often ventilator-associated pneumonia) pertaining to nosocomial infections (3) and community-acquired pneumonia (CAP) (4). S. aureus is a major pathogen of hospital-acquired pneumonia, whereas S. aureus CAP (SA-CAP) represents only 5% of CAP cases admitted to intensive care units in the United States (5) and 1 to 5.6% in Europe (6, 7). However, the prevalence of S. aureus in CAP has increased in recent decades, mainly due to the emergence of new lineages of methicillin-resistant Staphylococcus aureus (MRSA) that have become highly prevalent in the community, notably in the United States (8–10). Moreover, despite being infrequent, SA-CAP is a severe disease; mortality ranges from 20 to 44.5%, and there are significant age-dependent disparities (6, 9, 11–14). Deciphering the molecular mechanisms involved in host-pathogen interactions is therefore central to better understand SA-CAP physiopathology.

The human body, but more specifically the airway and the lungs, have broad defense mechanisms against pathogen invasion (15–20), at the forefront of which is the pulmonary epithelium (17, 19, 21–23), followed by professional immune cells (24, 25). How S. aureus is able to challenge those mechanisms and successfully colonize tissue and replicate is thus a central question. For nearly 3 decades, the role and mechanisms of action of S. aureus toxins, adhesins, proteases, and regulatory proteins have been extensively investigated and characterized *in vitro* (26–33). Nonetheless, the pathological impact of each of these virulence factors on pulmonary infections in humans is not fully understood. Historically, clinical evidence of their involvement, or at least association, in the occurrence and/or outcome of pneumonia has been assessed for a very limited number of them, namely, the alpha-toxin (Hla) in 1999 (34), the superantigenic toxic shock syndrome toxin 1 (TSST-1) in 2000 (35), and Panton-Valentine leucocidin (PVL) in 2002 (12). Since then, there has been growing evidence of the implication of these three toxins, as well as other virulence factors, at different steps of pulmonary infection.

This review follows step by step the mechanisms and virulence factors deployed by S. aureus during CAP, starting with the adaptation and invasion of the pulmonary tissue, followed by immune escape and the acute inflammatory response induced. Although pathogenesis is much more complex than this sequential description, this scheme was chosen for convenience of the review. The virulence factors involved in S. aureus CAP virulence with *in vitro* and/or *in vivo* evidence are summarized in Table 1. All *in vivo* data reported in this review were from animal models unless otherwise specified.

**TABLE 1:** Virulence factors implicated in S. aureus virulence during pneumonia with *in vitro* and/or *in vivo* evidence

Virulence factor	Infection step	Role/mechanism(s)^*b*^	Reference(s)^*a*^
*agr* system	Adaptation	Virulence regulation	**140**, **200**
Alpha-toxin (Hla)	Invasion	Pulmonary epithelial disruption	**59**, 100, **103**
	Host defense escape	Ciliary beat frequency impairment	98, 100, 107
		Macrophage digestion avoidance	130
	Mayhem in the lung	Cytokine production induction	101, 166, **167**, 168
Beta-toxin	Host defense escape	Ciliary beat frequency impairment	108
		Epithelial phagolysosome escape	113
	Mayhem in the lung	Cytokine production induction	163, 166
Biofilm	Adaptation	S. aureus aggregation and protection	62, **63**
ClfA/ClfB	Colonization	Adhesion factor	47, **48**
	Host defense escape	Macrophage phagocytosis inhibition	129
Cna	Colonization	Adhesion factor	201, 202
Complement-binding protein (Ecb)	Host defense escape	Complement inhibition	155
Delta-toxin (Hld)	Host defense escape	Epithelial phagolysosome escape	113
Enterotoxins B and C (SEB and SEC)	Mayhem in the lung	Abnormal T-lymphocyte activation	**170**, **171**
Fibrinogen-binding protein (Efb)	Host defense escape	Complement inhibition	**155**
Fibrinogen-binding protein (Fnbp)	Colonization	Adhesion factor	65
	Adaptation	Biofilm component	65, 68
Fur and iron acquisition	Adaptation	Metabolism adaption	**57**
IgG binding protein (Sbi)	Host defense escape	Complement inhibition	152, 153
IsdB	Adaptation	Adaptation to iron deprivation	29, **48**
Nuc	Host defense escape	NET DNA degradation	146, **147**, 148
Panton-Valentine leucocidin (PVL)	Host defense escape	Macrophage and neutrophil lysis	119, 122, 123, **138**
	Mayhem in the lung	Cytokine production induction	*12*, 120, 163
Phenol-soluble modulin α (PSMα)	Adaptation	Biofilm dispersion	86
	Host defense escape	Epithelial and macrophage phagolysosome escape	114
	Mayhem in the lung	Necroptosis induction	177
	Host defense escape	Neutrophil lysis	141, 142
Phevalin	Host defense escape	Epithelial phagolysosome escape	115
PhnD	Adaptation	S. aureus *a*ggregation	**59**
Staphylococcal protein A (Spa)	Invasion	Pulmonary epithelial disruption	70, 97
	Host defense escape	Ig binding	157
		Abnormal B lymphocyte activation and death	157, *158*, **159**
	Mayhem in the lung	Cytokine production induction	70, **160**
		Necroptosis induction	**177**
SElX	Host defense escape	Neutrophil phagocytosis inhibition	**143**, **144**
	Mayhem in the lung	Abnormal T-lymphocyte T activation	**143**
Serine protease SplA	Invasion	Mucine degradation	**96**
Staphopain A (ScpA)	Host defense escape	Surfactant protein A (SP-A) degradation	105
TSST-1	Mayhem in the lung	Cytokine production induction	35
		Abnormal T-lymphocyte T activation	**170**, **171**

a References for virulence factors implicated in pneumonia infection with animal models are highlighted in bold. References highlighted in italics present clinical evidence.

b NET, neutrophil extracellular trap(s).

## ADAPTATION AND INVASION

Very little is known about the mechanisms by which S. aureus induces lung infection in the absence of nasal carriage. However, it has been hypothesized that S. aureus reaches the lower respiratory tract from nasal colonization by air uptake during breathing by the host (1). Thus, nasal colonization by S. aureus and the associated increased risk of developing further infection has been thoroughly documented in cotton rats and mice (36, 37), as well as in clinical studies (38, 39), as reviewed by Sakr et al. (40) and by Kluytmans et al. (41); furthermore, nasal decolonization contributes to a decrease in deep S. aureus infections (42, 43). However, most studies were conducted on postoperative infections, therefore limiting our knowledge of the impact of S. aureus carriage on CAP occurrence. Nevertheless, the association between pneumonia and nasal colonization has been reported in several clinical studies (38, 41) and in two studies involving mouse models, in which previous nasal colonization increased the severity or the risk of developing pneumonia (37, 44). During nasal colonization, S. aureus upregulates several virulence factors (45), particularly adhesins such as clumping factor B (ClfB) (46, 47), wall teichoic acid (WTA) (36), and iron surface determinant A (IsdA), which is part of the iron acquisition pathway of S. aureus (29, 45). Recently, Yang et al. demonstrated that a vaccine against clumping factor A (ClfA) and IsdB, another protein from the iron acquisition pathway, reduces the severity of pneumonia in a mouse model after nasal injection (48), emphasizing the association of colonization factor expression with pneumonia. Overall, previous nasal colonization by S. aureus seems to be the main route of access to the lung. However, the mechanisms involved in the transition between the upper to the lower respiratory tract remain unclear.

### Adaptation to the lung environment.

S. aureus adapts to its new environment when it reaches the lungs and therefore modifies the expression of various factors in comparison with the nasal colonization state. Indeed, the lumen of the pulmonary tissue is poor in nutriments and metal ions, particularly iron (49), and is coated with pulmonary mucus (17, 50) that is composed of mucin (51) and surfactant proteins (18, 52). The low availability in nutriments reduces the overall energy metabolism of S. aureus with, notably, an upregulation of glycolysis and a downregulation of gluconeogenesis (53). Regarding iron uptake, S. aureus uses two main mechanisms, siderophores and heme acquisition by the Isd system or the heme transport system (Hts) (29, 54). The regulation of these mechanisms occurs predominantly through the ferric uptake regulator (Fur) (29), which is a central regulator for virulence adaptation and activation (55, 56). During lung infection, in the presence of low concentrations of iron, Fur promotes the Isd system (55, 57), as well as certain virulence factors, such as Hla and HlgC of gamma-hemolysin (57), and biofilm production through the upregulation of the *ica* operon (55, 58).

Aggregation and biofilm formation are pertinent mechanisms of bacterial adaptation to the lung environment, and S. aureus aggregates within a very short period of time (1 h after nasal inoculation), when it first interacts with the pulmonary epithelium (59). Among the S. aureus factors contributing to aggregation, PhnD, a phosphonate ABC transporter substrate binding protein, described in S. epidermidis for its contribution to biofilm (60), stabilizes the aggregate. Its depletion makes S. aureus more vulnerable to antibiotics and partially reduced lethality in a pneumonia mouse model (59). In addition to PhnD, in the sequence type 239 (ST239) MRSA lineage from Asia, a novel gene called *sasX* was identified as promoting nasal colonization and large bacterial aggregates (37). Further investigation on *sasX* has shown that its neutralization by specific antibodies reduces lung injuries *in vivo* (61). In addition to providing protection, this aggregate formation is the first step in the initiation of biofilm production (62). Biofilm allows both the protection and replication of S. aureus (33, 62, 63). Its composition depends on the maturation state, the environment, and the bacterium’s genetic background (64). However, several components are conserved, such as extracellular DNA, the microbial surface components recognizing adhesive matrix molecules (MSCRAMMs), and aggregation factors, as well as virulence factors that are mostly expressed in the last steps of the biofilm. Among the MSCRAMMs, most of them are already expressed during nasal colonization; these include fibrinogen-binding proteins (FnBPs), SdrC, and ClfB (26, 65–68). Among the other virulence factors potentially involved are protein A (Spa) (69, 70), either cell wall anchored or released into the extracellular milieu (71), as well as the CHIPS and SCIN proteins (72), both implicated in immune evasion (73, 74). Other compounds involved in biofilm formation were thoroughly described in a review by Paharik et al. (62).

Biofilm enhances resistance to phagocytosis and antimicrobial molecules (e.g., antibiotics), while also allowing S. aureus replication, leading to an increase in bacterial density and regulatory activity switching. At the beginning of lung colonization, only a few bacterial cells reach the lung epithelium, resulting in a low bacterial density and thus in an inactive quorum sensing (QS) regulatory system (*agr*) that leads to the low expression of its effector, RNAIII. In the absence of RNAIII, the transcriptional regulatory factor Rot represses the expression of several S. aureus virulence factors, including Hla, the PVL, and LukDE (75–77). This repression prevents a strong immune reaction in the early steps of the infection. Furthermore, Rot induces biofilm formation (78) and the expression of surface proteins (75, 79, 80) such as Spa, ClfB, and SdrC (66, 67). In the second step of colonization, biofilm formation allows S. aureus to replicate and to gradually increase its bacterial density, promoting the *agr* QS system. During its replication, S. aureus produces an accumulating amount of an autoinducing peptide (AIP), encoded by *agrD* of the *agrACDB* operon. Upon maturation and secretion via AgrB, AgrD AIP occurs in the form of a tailed thiolactone ring with autoinducing activity on the membrane protein, AgrC, belonging to a two-component regulatory system (TCS). The accumulation of AIP during exponential growth induces the autophosphorylation of AgrC in its cytoplasmic domain, leading to the activation of AgrA (the effector of the TCS), which in turn activates the *agrACDB* operon promoter in an autocatalytic circuit, as well as the divergent promoter P3 producing RNAIII (81, 82). RNAIII represses numerous cell wall-associated proteins and the global regulator Rot by an antisense RNase III-dependent mechanism (31, 83). Consequently, S. aureus expresses numerous virulence factors, such as PVL and Hla toxins (84, 85) and phenol-soluble modulins (PSM) (86, 87), which allow biofilm dispersion.

However, the *agr* system is not the only regulatory system involved in S. aureus virulence factor expression; SaeRS, a TCS which, unlike *agr*, is mainly activated by neutrophil signals (88, 89), also promotes the expression of toxins such as superantigens, hemolysins, and proteases (79, 88). In addition, a cytoplasmic regulator, SarA, activated during periods of metabolic stress or in the stationary growth phase, modulates virulence factor expression along with *agr* and SaeRS and upregulates the *agr* system (76, 90). Thus, S. aureus inaugurates lung colonization under the Rot regulation climax and later on, following bacterial replication, switches to a more aggressive state upon quorum sensing dependent activation of the *agr* system and SarA. This virulence state is exacerbated by the induction of the SaeRS system by the innate immune response (91). Specific mutations/variants of these regulatory systems, in particular for the *agr* system, have been related to more or less virulent strains in the context of pneumonia (92, 93).

This accurate adaption of S. aureus progressively enables it to reach, colonize, and then invade the pulmonary tissue, from the mucus to the extracellular matrix (ECM).

### Invasion.

**FIG 1:**
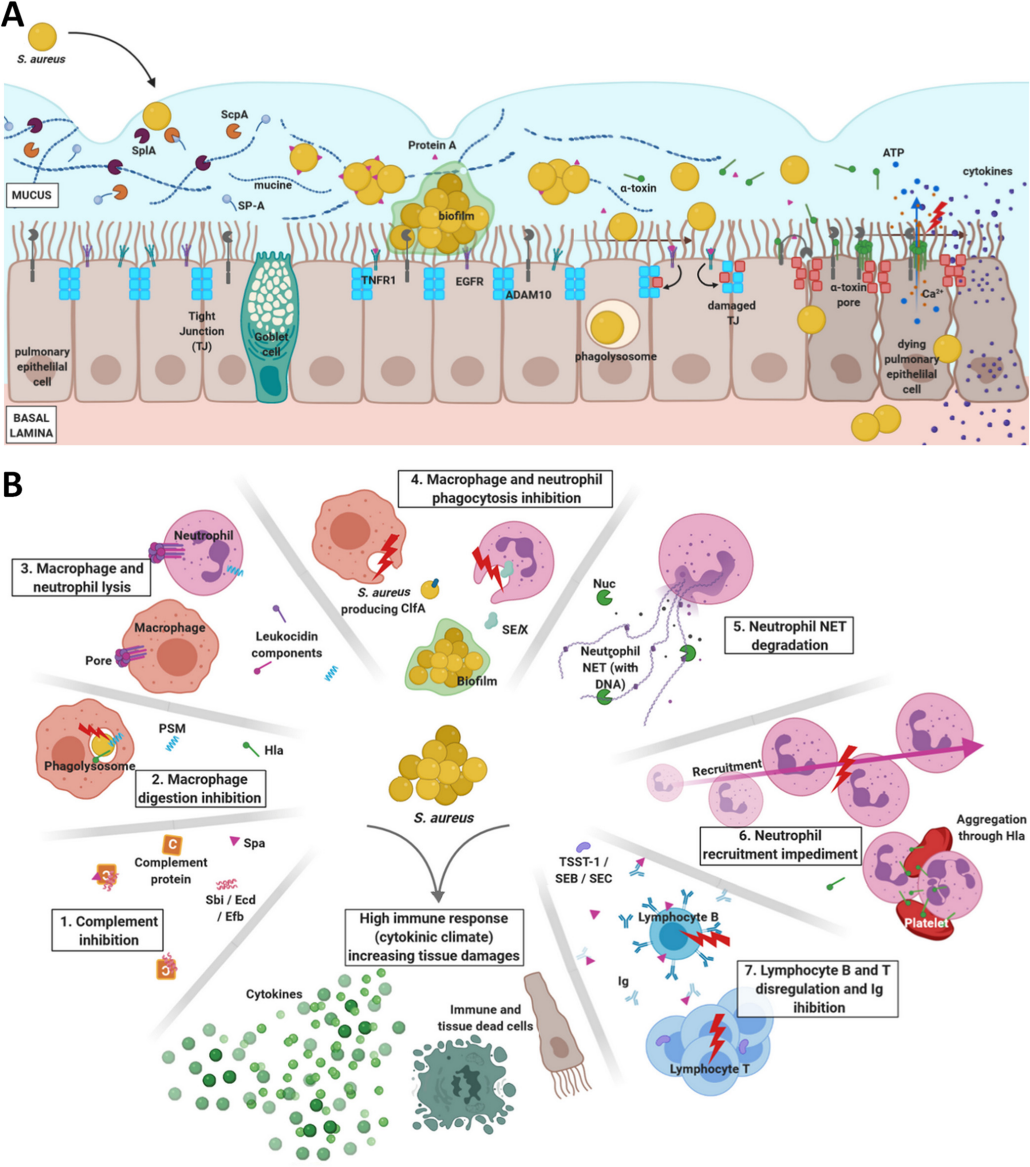
(A) S. aureus epithelial barrier invasion. S. aureus first crosses the mucus, which is made of mucine and surfactant protein A (SP-A). Both host proteins are degraded by serine protease SlpA and staphopain A (ScpA), respectively. S. aureus rapidly aggregates and cleaves staphylococcal protein A (Spa) from the cell wall. The aggregation leads to biofilm formation and quorum sensing *agr* regulation activation. Spa simultaneously interacts with the TNFR1 and EGFR host receptors on the pulmonary epithelial cells, initiating the disruption of the epithelial barrier. Upon reaching a bacterial density threshold, the biofilm is dissipated and the bacteria produce invasins, including the alpha-toxin (Hla). Hla pursues Spa pulmonary epithelium disruption through (i) disintegrin and metalloproteinase domain-containing protein 10 (ADAM10) hijacking that induces tight junction (TJ) degradation and (ii) pore formation, which lyses the epithelial cells. In addition, pore formation causes ATP and Ca^2+^ leaks that impede the ciliary beat frequency required for effective mucus clearance. Finally, epithelial cells are able to endocytose S. aureus, which can survive within the phagolysosome and escapes from it to induce the cell death. (B) S. aureus mechanisms to escape the host immune system, leading to harmful immune response. 1. Complement proteins can be trapped by Spa, Sbi, Ecd, and Efb, inhibiting S. aureus opsonization. 2. S. aureus can survive, replicate, and kill macrophages after its phagocytosis, thanks to Hla and PSMα. 3. Bicomponent leucocidin PVL, but also LukDE, LukAB, and HlgCB, can induce pore formation in neutrophil and macrophage membranes, leading to cell death. 4. S. aureus phagocytosis is prevented by biofilms and ClfA for macrophages and by biofilms and SElX for neutrophils. 5. The nuclease Nuc can degrade neutrophil NET DNA to avoid trapping. 6. SCIN, CHIPS, Spa, and Sbi are proteins capable of inhibiting neutrophil recruitment. By aggregating platelets and neutrophils, Hla can also impede neutrophil recruitment at the infection location. 7. Both lymphocytes are targeted—B cells by Spa and T cells by TSST-1, SEB, and SEC. The interactions between the toxins and the lymphocytes dysregulates lymphocyte activation and replication. Spa can also interact with Ig, preventing its interaction with the bacteria. All of these mechanisms increase the immune response, generating acute inflammation that damages the pulmonary tissue, in addition to S. aureus damage. PSM, phenol-soluble modulin(s); NET, neutrophil extracellular trap(s). Figure created with BioRender.com.

To colonize the tissue, S. aureus first passes through the mucus and then through the tight junctions (TJs) of lung epithelial cells (Fig. 1A). The mucus is produced by the goblet cells to protect the epithelium by trapping microorganisms and is therefore the first host rampart. It is composed of water, ions, lipids, surfactant proteins (18, 94), and highly glycosylated proteins such as mucins (95). To get through the mucus, S. aureus targets its major component, mucin, using the serine protease SplA, which cleaves mucin-16 from the human pulmonary cell line (Fig. 1A). A mutant with *splA* deleted displayed reduced lung invasion *in vivo* (96). Subsequently, Spa and Hla initiate the disruption of the epithelial barrier (Fig. 1A). Spa destabilizes the epithelial barrier through its interaction with the EGFR and TNFR1 receptors on the cell surface (70). Spa interaction induces the activation of the RhoA/ROCK/myosin light chain (MLC) eukaryotic cell pathway, which results in the disruption of TJ of the epithelium (97). Hla also plays an important role in tissue invasion, since it can participate in pulmonary epithelium disruption and destruction. Hla is a pore-forming toxin (PFT) (98) that targets the disintegrin and metalloproteinase domain-containing protein 10 (ADAM10) (99), which is expressed at the epithelium surface. The interaction between ADAM10 and Hla leads to numerous molecular reactions, starting with the oligomerization of Hla proteins to form an heptameric β-barrel pore in the membrane (98). Pore formation leads to the release of ions, notably Ca^2+^, which allows not only TJ disruption (59, 100) but also cell lysis (Fig. 1A) (59, 99, 101, 102). Moreover, the recruitment of ADAM10 by Hla hijacks its function; therefore, ADAM10 participates in TJ degradation by cleaving it through its enzymatic activity (Fig. 1A) (103). Overall, Hla and Spa are essential S. aureus weapons when infecting the host lung, since they allow S. aureus to pass from the lung lumen to the ECM (Fig. 1A). Although the *agr* system is activated, repressing MSCRAMM expression, the remaining MSCRAMMs at the surface of S. aureus allow strong interaction between S. aureus and the ECM (104).

In summary, S. aureus adapts to its new environment by regulating specific metabolic and virulence pathways. Subsequently, during its passage from the mucus of the lumen to the ECM, S. aureus evades numerous host defense mechanisms provided by the epithelium and the host immune system.

## HOST DEFENSE ESCAPE

The lungs are confronted by numerous microorganisms, from the commensal population to invaders. Different cells and defense mechanisms are deployed by the host to prevent invasion from pathogenic microbes. The pulmonary epithelium, along with its mucus layer, forms the first rampart, followed by the immune system, and S. aureus has developed toxins and enzymes to escape these defense mechanisms.

### Pulmonary epithelium.

Within the mucus, the surfactant components are proteins with antimicrobial activities that initiate the killing of entrapped microorganisms (52) before their physical expulsion by the mucociliary escalator (19, 94). S. aureus simultaneously targets mucins with SplA (Fig. 1A) and surfactants with staphopain A (ScpA), a hydrolyzing enzyme that degrades surfactant protein A (SP-A, not to be confounded with Spa [staphylococcal protein A]) (Fig. 1A) (105), an airway immune defense effector (52). The deterioration of SP-A enables S. aureus to aggregate and adhere to the pulmonary epithelium (105).

The ciliary beat of the pulmonary epithelial cells allows the clearance of mucus-embedded microorganisms. To avoid its elimination, S. aureus inhibits the mucociliary escalator in several ways. Pore formation by the oligomerization of Hla in the membrane of epithelial cells allows the release of ions, notably Ca^2+^ (98, 100), and the leak of ATP in the extracellular medium (106), both of which can modify the ciliary beat frequency (Fig. 1A) (107). In addition, the synergistic action of the beta-toxin with Hla between 8 and 12 h after their contact with the epithelial cells drastically decreases the ciliary beat required for effective mucus clearance (34, 108).

Finally, although the pulmonary epithelial cells are nonspecialized phagocytes, they can capture S. aureus. It is as yet unclear whether the epithelial cells actively phagocytose S. aureus, if it is S. aureus that invades the epithelial cell, or if it is a combination of both. Indeed, epithelial cells can internalize bacteria (109–111), a mechanism mediated in part by the efflux pump Tet38 of S. aureus (Fig. 1A) (111, 112). S. aureus is able to enter pulmonary epithelial cells through the interaction between the Tet38 efflux pump and CD36 eukaryotic cell membrane protein in A549 cells (lung epithelial cell line) (112). Following internalization, S. aureus rapidly escapes from the phagolysosome (2 h) and induces cell death via several effectors, namely PSMα, PSMβ, Hld, beta-toxin (111, 113, 114), and a nonribosomal peptide (phevalin) previously identified in Streptomyces (115, 116) and which is required for full virulence *in vivo* (115).

Altogether, S. aureus possesses several toxins and enzymes that allow the bacteria to penetrate through the mucus and the epithelial barrier. Although the pulmonary epithelium is a major line of defense, the host also possesses other cells and mechanisms to protect itself, notably the immune response. However, even these defenses can be defeated by S. aureus.

### Macrophages.

Alveolar macrophages are the resident macrophages of the pulmonary tissue and the second line of defense when pathogens succeed in reaching and crossing the epithelium (20). Their major function is to phagocytose the pathogens or their debris, a function that is impaired by S. aureus through three major mechanisms.

The first mechanism that allows S. aureus to avoid macrophage phagocytosis is to destroy the macrophages using pore-forming toxins (PFT), also known as leucocidins. Leucocidins target specific cells through specific receptors at their membrane surfaces to induce pore formation, followed by cell death (Fig. 1B) (32). Five leucocidins, namely, LukAB (LukGH) (117), LukDE (118), HlgAB, HlgCB, and PVL (119, 120), target macrophages after their interactions with their receptors, namely, CD11b for LukAB, CCR5, and CXCR1/2 receptors for LukDE (118, 121), CCR2 and CXCR1/2 for HlgAB, C5aR1/R2 for HlgCB, and C5aR1/R2 and CD45 for the PVL (119, 122, 123). Except for the PVL, whose impact on pneumonia severity has been highlighted by several studies, including clinical ones (12, 14, 124–128), the roles of LukAB, LukDE, and HlgAB/CB have not yet been elucidated.

The second way for S. aureus to a avoid phagocytosis is by protecting itself by hiding and forming agglomerates inside the biofilm (Fig. 1B) (63). In this context, ClfA, which is expressed during biofilm formation, as mentioned in the “Adaptation to the lung environment” paragraph, impairs macrophage phagocytosis (129). Although there is no direct evidence of ClfA involvement with alveolar macrophages, Yang et al. reported that immunity against ClfA reduces pneumonia severity in mice (48).

Finally, the third mechanism to counter phagocytosis is the capacity of S. aureus to survive within macrophages. The activation of the NLRP3 inflammasome by Hla leads to the recruitment of mitochondria away from the phagosome, thus preventing the digestion of the bacteria inside the phagolysosome (Fig. 1B) (130). In addition, two studies have reported the ability of S. aureus to survive, replicate, and finally kill macrophages (131), in particular due to PSMα (114).

### Neutrophils.

Neutrophils are professional antimicrobial cells that use phagocytosis, degranulation, antimicrobial proteins, and neutrophil extracellular traps (NET) to clear pathogens from infected tissues (20, 24). As a result, human neutrophils are one of the main targets of S. aureus toxins (132, 133).

First, as neutrophils are nonresident immune cells, S. aureus can impair neutrophil recruitment by the expression of CHIPS and SCIN factors, which are chemotaxis inhibitors (Fig. 1B) (24, 73, 74) and which are part of an “immune evasion cluster” present in human strains but generally not in bovine strains (134). Staphopain A can also inhibit the chemotaxis of neutrophils and their activation (Fig. 1B) (135); however, the role of these three proteins in the course of pneumonia in human remains to be confirmed. Another possible mechanism preventing the proper localization of neutrophils at their target site is the aggregation of both platelets and neutrophils induced by Hla, thus disturbing their recruitment, a mechanism that may occur in the lung (Fig. 1B) (136).

Second, S. aureus is able to kill neutrophils, largely by the activity of PFT. The role of PVL is predominant in neutrophil lysis (137), notably demonstrated in rabbits and humanized mouse models of pneumonia (Fig. 1B) (124, 125, 138). The bicomponent leucocidins like HlgCB, which targets the same receptors as PVL, LukDE, and LukAB, have not been experimentally demonstrated as key players in S. aureus pneumonia so far. Conversely, the degradation products of PSM-lysed neutrophils (139) are responsible for lung injuries in mice (140). Moreover, PSMs can kill neutrophils upon S. aureus phagocytosis (141), and PSMα3 synergizes with PVL to kill neutrophils (Fig. 1B) (142).

Another way to inhibit the function of neutrophils is to block their phagocytosis. Superantigen exoprotein (SAg) is a group of toxins that includes the toxic shock syndrome toxin 1 (TSST-1) and 18 staphylococcal enterotoxins (SE). These are responsible for polyclonal T-cell receptor (TCR) activation, leading to massive T-cell expansion and cytokine secretion. In 2011, a staphylococcal enterotoxin-like protein, SElX, encoded in the core genome, was identified as being implicated in S. aureus virulence in a rabbit model of pneumonia (143). Recently, one of its mechanisms was characterized; unlike the other SAg, this mechanism does not involve interaction with T cells, but it inhibits neutrophil phagocytosis (Fig. 1B) (144), leading to increased mortality in a pneumonia rabbit model.

Finally, NET are a well-organized and structured combination of DNA and cytosolic proteins that entrap pathogens in order to kill them and prevent their dissemination (145). DNA is one of the major components of NET; therefore, the secretion of Nuc, a nuclease produced by S. aureus, is very relevant means of escaping from NET (Fig. 1B) (146). In a murine pulmonary infection model, secretion of Nuc in the lung decreased S. aureus clearance and increased mortality (147). S. aureus is also able to counteract NET by other mechanisms, such as the conversion of NET into deoxyadenosine, which induces immune cell death (148). However, this mechanism was explored in kidney abscess in a mouse model and not in pneumonia.

### Opsonization and humoral response.

Phagocytosis by specialized cells such as macrophages and neutrophils can be induced by the recognition of bacterial cell walls or opsonins, such as complement proteins or antibodies (mostly IgG) (149, 150), that target both cell wall-associated proteins and exotoxins (151). In addition to its direct action on phagocytic cells, S. aureus targets the complement or antibody-mediated opsonization pathways.

S. aureus secretes proteins that inhibit complement proteins, such as Sbi, which complexes complement proteins (152, 153), and Ecb (also known as Ehp), which inhibits the convertase activity of the C3 complement protein, as does Efb through its C terminus (154). Deletion mutants of Ecb and Efb proteins are reported to provoke reduced mortality *in vivo* (Fig. 1B) (155). Other proteins, such as SCIN and CHIPS, which inhibit neutrophil chemotaxis through the complement, and Spa, have been reported to impede the complement; nonetheless, their involvement in pneumonia through complement inhibition has yet to be proved (156).

Spa is important in S. aureus virulence in the context of pneumonia, as mentioned previously, and, in addition to epithelium disruption, Spa impedes the humoral response. It is cleaved and released in to the extracellular environment (71) and is able to bind Ig and prevent interaction with the target epitope (157). Furthermore, it interferes in lymphocyte B activation and proliferation, leading to (i) the reduction of phagocytosis of S. aureus (157), (ii) antibody production impediment (158), and (iii) disordered activation, finally leading to the death of the B cells (Fig. 1B) (159). These mechanisms have not yet been studied in the context of pneumonia; however, due to the significant interaction between Spa and the variable heavy 3 (VH3)-type B cell receptors, these phenomena would very likely occur in the lungs.

Overall, S. aureus has developed a wide range of proteins to inhibit and escape host defenses, including the immune system. Nevertheless, the presence of S. aureus, and the lysis and dysregulation of epithelial and immune cells, lead to an acute inflammatory response. This acute inflammation can still be an advantage for S. aureus, by contributing to overall tissue damage.

## MAYHEM IN THE LUNG

### Cytokine production.

The acute inflammatory response first results in the accumulation of cytokines produced by the lung epithelial cells (Fig. 1B). More specifically, during S. aureus infection, the interaction between S. aureus and the Toll-like receptor 2 (TLR2) of the epithelial cell induces the NF-κB pathway, and the production of proinflammatory cytokines and tumor necrosis factor (TNF) (156). Furthermore, via its interaction with TNFR1 (70, 160), Spa primes the secretion of interleukin-8 (IL-8) and of IL-16, an immune cell chemoattractant (161), which is responsible for lung damage *in vivo* (160). This phenomenon is amplified by the activation of EGFR, which increases the availability of TNFR1 at the cell surface (162). Moreover, the interaction between Hla and ADAM10 induces the secretion of IL-1β by the epithelium; the knockout of ADAM10 in mice protects against lethal S. aureus pneumonia through the reduction of IL-1β production (101). The PVL toxin also leads to IL-1β production by macrophages, acting in a paracrine manner to trigger IL-8 secretion by the epithelial cells (163). Finally, TSST-1 also induces the production of TNF and IL-8 by the pulmonary epithelium and thus promotes inflammation (35). Taken together, the epithelium initiates a strong cytokinic response in order to mobilize the immune system.

The pulmonary epithelial cells produce cytokines when in contact with S. aureus toxins, as do the immune cells via the NLRP3 inflammasome pathway (Fig. 1B). The inflammasome is an intracytoplasmic multiprotein complex activated by cell stresses or infections and is responsible for the release of proinflammatory cytokines, including IL-1β (164, 165). Beta-toxin, gamma-hemolysin (25, 163, 166), PVL (25, 120, 163), and Hla (166–168) trigger the NLRP3 inflammasome in macrophages, monocytes, and neutrophils, resulting in proinflammatory IL-1β and IL-18 secretion that is responsible for necrotic injuries and severe pneumonia *in vivo* (167). The inhibition of the inflammasome in the pneumonia mouse model results in a decrease of cytokines in the pulmonary tissue and therefore in decreased lung injuries (169). In addition to inflammasome activation in immune cells, most of these toxins, such as PVL and gamma-hemolysin, can lyse macrophages and neutrophils (119, 122), leading to an involuntary release of cytokines and exacerbating local inflammation.

### Lymphocyte response dysregulation.

In addition to this cytokine storm, S. aureus impacts several other immune mechanisms, including the activation of lymphocytes (Fig. 1B). As mentioned above, SAg TSST-1, along with the enterotoxins SEB, SEC, and SElX, induces nonspecific T-lymphocyte activation and proliferation and, as a result, increases lung damage (143, 170). This polyclonal activation of T cells participates in the cytokinic storm, as T cells promote inflammation through cytokine release. These deleterious effects are circumvented by antibodies against TSST-1, SEB, and SEC in rabbit models (171). In addition, Parker et al. have demonstrated that this T-cell activation contributes to S. aureus pathogenicity by increasing lung damage (172). Therefore, the inhibition of abnormal T-cell activation might be a therapeutic option for future development.

B lymphocytes, implicated in antibody responses, are also targeted, especially by Spa. The latter first monopolizes the immunoglobin response by promoting anti-Spa immunoglobins produced by plasma cells and B cells, instead of immunoglobins against other secreted toxins, such as Hla or PVL (158). Second, Spa, by its ability to interact with B cell receptors, reduces their proliferation, but it predominantly induces B cells apoptosis after 36 to 48 h via the caspase pathway (159) via Spa shedding from the bacterial surface (71). As immunotherapies provide good evidence of efficacy to reduce the risk of occurrence and severity of S. aureus pneumonia in animals (173), and also in humans (174), the impedance of B cells by S. aureus may contribute to the persistence of infection.

Thus, both types of lymphocyte are impaired by S. aureus, with, on the one hand, an increase of inflammation by the T lymphocytes and, on the other hand, an inefficient humoral response dependent on B lymphocytes, allowing persistent S. aureus infection.

### Necroptosis induction and efferocytosis inhibition.

Necroptosis is a cellular suicide mechanism used by microorganism-infected cells to prevent the replication and spread of the intruder (175). It leads to the activation of a proinflammatory pathway (IL-6, TNF, IL-1α, and IL-1β) that recruits phagocytes to clear debris after cell death and phagocytose dying cells (efferocytosis). The latter mechanism is important for the resolution of inflammation and tissue integrity restoration (176).

S. aureus is able to impact both mechanisms. First, it increases necroptosis, leading to acute cytokine release through secretion of toxins. Indeed, Hla, LukAB, and PSM participate in necroptosis mechanism induction by activating RIP1/RIP3/MLKL signaling in macrophages. Necroptosis impairment or inhibition of these toxins in the pneumonia mouse model decreases S. aureus virulence and improves its clearance (177). Regarding efferocytosis, to date only Hla has been reported as an inhibitor; by interacting with alveolar macrophages, it reduces their ability to phagocytose dying neutrophils (178). In 2014, Greenlee et al. demonstrated that when phagocytosed by neutrophils, S. aureus survives in the phagolysosome and decreases the efferocytosis of neutrophils by macrophages. In addition, S. aureus increases the production of cytokines by macrophages, exacerbating inflammation in the tissue (179). However, the pathophysiological impact of this phenomenon has not yet been assessed in any disease models, and more studies are required to further understand the manipulation of these mechanisms by S. aureus.

Other cell death mechanisms are also impeded or diverted and were recently reviewed by Grousd et al. (180).

Taken together, S. aureus pneumonia can lead to severe outcomes due to the tissue necrosis induced by S. aureus itself, but also to immune-driven inflammation. The reduction of this inflammation is one way to prevent lung tissue damage. This has been demonstrated in pneumonia mouse models, in which cytokine production was hindered by the inhibition of NF-κB signaling (181), NLRP3 inflammasome inhibition (169), and IL-1R signaling (182). Another way is to inhibit S. aureus toxins, notably by using passive immunotherapy approaches, such as neutralization with antibodies targeting Hla, PVL, HlgACB, and LukDE. This strategy has been demonstrated to be effective to protect animal models from S. aureus pneumonia (126, 127).

## DISCUSSION AND PERSPECTIVES

S. aureus CAP are rare but severe infections with a high rate of lethality (9, 13). We describe here an arsenal of virulence factors produced by S. aureus that are implicated in its adhesion and adaptation to, as well as invasion of, the lung epithelium. The adaptation includes profound metabolic changes of the bacterium, notably in response to iron and nutrient limitations (53). However, most studies have assessed the impact of a given virulence factor using isogenic mutants or specific inhibitors, and therefore by comparing the presence/absence of the protein studied. These approaches omitted the notion of protein abundance. Indeed, most virulence factors belong to the bacterial core genome, and thus *in vivo* their impact on the host should reasonably depend on their level of expression. Only a few studies focusing on S. aureus pneumonia have investigated this parameter. Nevertheless, they offer new perspectives in the investigation of S. aureus virulence. For instance, in 2013, a link was established between mortality in the rabbit pneumonia model and Hla and PVL concentrations in lung samples (183). In humans, a severe outcome in ventilator-associated pneumonia was associated with higher Hla production *in vitro* (184). Quantitative approaches assessing the full spectrum of S. aureus virulence factors remain essential to fully understand the multifactorial nature of bacterial pathogenesis.

An additional aspect that is often discussed but difficult to explore is research using human samples and associated clinical data. As described in this review, several experimental models (cell culture and animal models) have been used to study interactions between S. aureus and the lung environment. Although animal models tend to mimic the highly complex human physiopathology during pneumonia as well as possible, they remain simplistic, with obvious biases such as species-specific receptor polymorphisms, leading to great variations in susceptibility to a given toxin depending on the animal species (32). This phenomenon has been perfectly deciphered for PVL, for which, surprisingly, our closest relative (nonhuman primate) was mostly resistant to the toxin (185). Therefore, caution is necessary when drawing conclusion for human based on cellular or animal models describing S. aureus infection mechanisms.

One aspect not explored in this review is the complexity of the lung microbiota, which may impact both the transition from colonization to invasive infection and S. aureus virulence. These aspects were more explored in other settings, such as cystic fibrosis patients or hospital-acquired pneumonia, but are poorly defined in the context of CAP (186, 187). Finally, we did not develop the impact of other clinical conditions on CAP occurrence or severity, such as host comorbidity factors and the role of previous viral infection damaging the pulmonary epithelium. For example, several clinical and experimental studies have reported a consistent link between previous influenza infections and the severity of SA-CAP (44, 188–197). Influenza infections induce a switch in S. aureus to a more virulent status (44), and the virus damages the host epithelium (188, 191, 194) but also promotes S. aureus intrusion and adhesion to the pulmonary tissue (189, 192, 195). Another means for S. aureus to reach the lung is via the hematogenous route in the course of bacteremia. Clinical observations suggest that in the case of pneumonia initiated by the air route of infection only one lobe can be infected, whereas multilobar infections can be observed upon bacteremia. However, there is no experimental evidence suggesting that specific virulence factors are associated with one or the other route of infection; yet, it is of interest to note that Hla was shown to impact the severity of pneumonia by the hematogenous route in rabbit. Conversely, epidermal differentiation inhibitor B (EdinB), initially described as a potential virulence factor in skin infection by impairing the maturation of keratinocytes, was shown to increase S. aureus translocation to the blood in the course of pneumonia in mice (198, 199); however, this remains to be fully investigated.

S. aureus is able to cause severe infections, notably in the lung, through the production of an array of toxins and proteins. These virulence factors are highly efficient in counteracting the host’s defense, including the immune system, which is even used to the advantage of S. aureus. With the emergence of new strains that possess newly discovered genes or accessory genes encoding toxins, such as the MRSA USA300 clones or the ST239 lineage that are implicated in CA pneumonia infection, better understanding of S. aureus virulence mechanisms is required to develop new therapeutic strategies.

In conclusion, the present review illustrates how it is the association of several virulence factors at specific infection steps, and the host response to these factors, that leads to severe staphylococcal pneumonia.
